# Euthanasia and other end-of-life decisions: a mortality follow-back study in Belgium

**DOI:** 10.1186/1471-2458-9-79

**Published:** 2009-03-09

**Authors:** Lieve Van den Block, Reginald Deschepper, Johan Bilsen, Nathalie Bossuyt, Viviane Van Casteren, Luc Deliens

**Affiliations:** 1 Vrije Universiteit Brussel, End-of-Life Care Research Group, Laarbeeklaan 103, 1090 Brussels, Belgium; 2 Vrije Universiteit Brussel, Department of General Practice, Laarbeeklaan 103, 1090 Brussels, Belgium; 3 Ghent University, Bioethics Institute, Blandijnberg 2, 9000 Ghent, Belgium; 4 Scientific Institute of Public Health, Department of Epidemiology, Juliette Wytsmanstraat 14, 1050 Brussels, Belgium; 5 Department of Public and Occupational Health, EMGO Institute for Health and Care Research, VU University Medical Centre, Amsterdam, the Netherlands

## Abstract

**Background:**

This study compares prevalence and types of medical end-of-life decisions between the Dutch-speaking and French-speaking communities of Belgium. This is the first nationwide study that can make these comparisons and the first measurement after implementation of the euthanasia law (2002).

**Methods:**

We performed a mortality follow-back study in 2005–2006. Data were collected via the nationwide Sentinel Network of General Practitioners, an epidemiological surveillance system representative of all Belgian GPs.

Weekly, all GPs reported the medical end-of-life decisions among all non-sudden deaths of patients in their practice. We compared the northern Dutch-speaking (60%) and southern French-speaking communities (40%) controlling for population differences.

**Results:**

We analysed 1690 non-sudden deaths. An end-of-life decision with possible life-shortening effect was made in 50% of patients in the Dutch-speaking community and 41% of patients in the French-speaking community (OR 1.4; 95%CI, 1.2 to 1.8). Continuous deep sedation until death occurred in 8% and 15% respectively (OR 0.5; 95%CI, 0.4 to 0.7). Community differences regarding the prevalence of euthanasia or physician-assisted suicide were not significant.

Community differences were more present among home/care home than among hospital deaths: non-treatment decisions with explicit life-shortening intention were made more often in the Dutch-speaking than in the French-speaking community settings (OR 2.2; 95%CI, 1.2 to 3.9); while continuous deep sedation occurred less often in the Dutch-speaking community settings (OR 0.5; 95%CI, 0.3 to 0.9).

**Conclusion:**

Even though legal and general healthcare systems are the same for the whole country, there are considerable variations between the communities in type and prevalence of certain end-of-life decisions, even after controlling for population differences.

## Background

Several studies have shown that death is often preceded by a medical end-of-life decision with possible or certain life-shortening effect such as non-treatment decisions, intensification of symptom alleviation, euthanasia, physician-assisted suicide, or the use of lethal drugs without the patient's explicit request [1-20]. Both the incidence and the characteristics of the decision-making process have been found to differ between countries [4,7,14,16,20,21]. However, even when using the same methodology in international comparative research, interpretation of differences between countries is very difficult since totally different legal and healthcare systems need to be compared.

Belgium is a unique country in which to study variations in end-of-life decision-making. It is one country, where legal and general healthcare structures (e.g. social security system, accessibility criteria for healthcare provision, general healthcare budget) are defined and regulated at national level, but where two language communities can be differentiated: the Dutch-speaking northern community (60% of the population) and the French-speaking southern community (40%), both separately responsible for policy concerning culture, education, and certain aspects of healthcare (e.g. prevention, organisation of healthcare) [22-26]. The investigation of whether variations in end-of-life decisions occur under these circumstances can give important insights into the factors underlying them.

Furthermore, Belgium is one of the three countries in the world with a law on euthanasia [22]. Notification of euthanasia cases is legally obligatory. However, despite having the same legal system, it is remarkable that 82% of all cases reported to the Federal Evaluation and Control Committee on Euthanasia in 2005 and 2006 came from Dutch-speaking physicians, while only 18% came from French-speaking physicians [27-29]. The largest proportional difference was found for patients who died at home or in care homes (92%-8%) compared to hospital settings (81%-19%) [29]. Because this cannot be explained by differences in population size, finding empirically-supported explanations has become an issue of considerable medical and societal debate. It also raises questions concerning differences in other end-of-life decisions in homecare and hospital settings.

Previous reports concerning end-of-life decision-making in Belgium could not make comparisons between the two communities. Due to the absence of recent death certificates in the French-speaking community which would identify physicians, previous reports only cover the Dutch-speaking community [3,4]. Additionally, these figures date from the time before the euthanasia law was implemented in 2002 [3,4,22].

The objective of this study is to investigate possible differences in prevalence and types of end-of-life decisions between the Dutch-speaking and French-speaking communities of Belgium after the implementation of the euthanasia law. We focus on three research questions. How prevalent are different types of end-of-life decisions in Belgium in 2005–2006? Are there differences between the two communities in the prevalence of different types of end-of-life decisions or in the decision-making process? Are these differences present in homecare settings as well as in hospital settings in both communities?

## Methods

### Design

Because general practitioners (GPs) are pivotal healthcare providers in Belgium and 95% of the population, including care home residents, have a regular GP [30], we collaborated with the existing nationwide Belgian Sentinel Network of General Practitioners to study a population-based sample of deaths in Belgium. This network, operational since 1979, has proved to be a reliable surveillance system for a wide variety of health-related epidemiological data [31-33]. In 2005 and 2006, it consisted of respectively 181 and 174 regularly participating practices, representative of all 10,578 GPs in Belgium in terms of age, gender and region and covering 1.75% of the total Belgian patient population [31,34].

In 2005 and 2006, this network participated in a two-year nationwide mortality follow-back study, the senti-melc study, which was designed to **m**onitor **e**nd-of-**l**ife **c**are via this **senti**nel GP Network [33]. The GPs from the sentinel network registered weekly all deaths of patients in their practice older than one year, including deaths that occurred in hospital settings. They registered each death immediately it occurred or immediately on being informed, using a standardised form. If patients died in a hospital setting, GPs were informed by the hospital specialists.

Anonymity of the patient and the physician was preserved. Every patient that is registered within the network received an anonymous reference from the GP him/herself, and all identification codes of the GPs were replaced in the data files with anonymous codes during data cleaning. Details of this procedure are described elsewhere [33]. The study protocol was approved by the Ethical Review Board of Brussels University Hospital.

### Measurements

The first part of the registration surveyed the patient characteristics of all registered patients and whether or not death had occurred "suddenly and totally unexpectedly" [4,35]. For patients who died non-suddenly, a second part surveyed end-of-life decision-making. Following previous studies [3,4] we studied 4 types of end-of-life decisions with a possible or certain life-shortening effect, taken by the responding GP him/herself or by another physician: (1) non-treatment decisions (taking into account a possible hastening of death or with the explicit intent to hasten death); (2) intensifying alleviation of pain or other symptoms (taking into account or co-intending the hastening of death); (3) administering (i.e. euthanasia), supplying or prescribing (i.e. physician-assisted suicide) drugs with the *explicit *intention of hastening death on the patients' explicit request; and (4) administering drugs with the *explicit *intention of hastening death without explicit patient request. Concerning the decision-making process, we investigated whether the decision was taken after discussion with the patient, on his/her explicit request, and whether the patient was competent to make decisions. The GPs were instructed to fill in these questions only if one of the questions on end-of-life decisions was answered "yes".

Finally, we also asked whether or not continuous deep sedation was performed, defined as a patient being deeply and continuously sedated or in a coma until death, by means of e.g. benzodiazepines or barbiturates (continuous deep sedation) and if so, whether artificial food or fluid was administered (palliative sedation) or not (terminal sedation). The wording of all questions and classification of practices was identical to previous incidence studies although these used another research design [1-4,10,17].

The instrument was first developed in Dutch and then translated into French via forward-backward translation. It was tested and slightly adapted after an extensive pilot study [33]. Several control measures were used to ensure data quality: automatic follow-up and possible telephone contact with the GP to prevent missing data for key variables (e.g. euthanasia), data-entry with consistency, range and skip checks, and double data-entry.

Detailed descriptions of the SENTI-MELC study's methodology, the questionnaire and first set of results have previously been reported [33,36-38].

### Analysis

We calculated the prevalence of end-of-life decisions for Belgium using binominal 95% confidence intervals (exact method). To analyse differences between the communities in Belgium, we compared cases from physicians in the Dutch-speaking northern part with cases from physicians in the French-speaking southern part. We used logistic regression analysis to calculate odds-ratios correcting for differences in patient characteristics. Interaction effects between community and each patient characteristic were systematically evaluated. We studied differences between the communities in total, as well as for homecare and hospital settings separately subdivided on the basis of the patient's place of death. Differences in decision-making characteristics were studied using Fishers' Exact Tests. All analyses were performed using StatXact6 (Cytel Studio, Cambridge, MA) and SPSS14.0 (SPPS Inc, Chicago, IL).

## Results

### Study population

The GPs reported 2690 deaths, of which 64.3% were non-sudden. Forty-one cases (2.4%) were excluded from the SENTI-MELC study because of missing data. Thus, the results are based on 1690 non-sudden deaths, described in Table 1. Age, sex, educational level, cause of death and place of death for non-sudden deaths did not differ between the two communities (p > .05). Age, sex and place of death for non-sudden deaths in Flanders (N = 1032) could be compared with non-sudden deaths identified in a previous study on end-of-life practices representative of all deaths in this same part of the country (n = 2128) [4]. There were no significant differences for these characteristics (binomial 95% CI, exact method).

**Table 1:** Characteristics of the study population of non-sudden deaths by community in Belgium

		Dutch-speaking community N = 1032		French-speaking community N = 658		Total for Belgium N = 1690	
		
Patient characteristics^a^		N	%	N	%	N	%
Age^b^	1–64 y	118	11.6	81	12.5	199	12.0
	65–84 y	574	56.7	358	55.2	932	56.1
	85 y+	321	31.7	209	32.3	530	31.9
Sex	Male	526	51.0	313	47.6	839	49.6
	Female	506	49.0	345	52.4	851	50.4
Educational level^b^	Elementary or lower	431	46.3	235	40.9	666	44.3
	Lower secondary	245	26.3	180	31.3	425	28.2
	Higher secondary	173	18.6	113	19.7	286	19.0
	Higher education	81	8.7	47	8.2	128	8.5
Cause of death^b^	Cardiovascular dis.	136	13.3	101	15.5	237	14.2
	Malignancies	455	44.6	270	41.5	725	43.4
	Respiratory dis.	96	9.4	61	9.4	157	9.4
	Dis. nervous system^c^	110	10.8	82	12.6	192	11.5
	Other	223	21.9	137	21.0	360	21.5
Place of death	Home or with family	226	21.9	177	26.9	403	23.8
	Care home	276	26.7	176	26.7	452	26.7
	Hospital	421	40.8	244	37.1	665	39.3
	Palliative care unit	109	10.6	61	9.3	170	10.1

^a ^**p > .05 **for all characteristics (Fishers' Exact test)

^b ^Missing values for age n = 29; for level of education n = 185; for cause of death n = 19

^c ^including stroke

### Frequencies of end-of-life practices

In 47% of all patients who died non-suddenly, death was preceded by an end-of-life decision with possible life-shortening effect in Belgium (Table 2). This involved euthanasia/physician-assisted suicide in 1.3% of cases, life-ending drug use without the patient's explicit request in 1.6%, intensified symptom alleviation in 28%, and non-treatment decisions in 16% of cases. Continuous deep sedation was registered in 11% of cases in total, for which 6% and 5% respectively involved the administration and forgoing of artificial food or fluid.

**Table 2:** Frequency of end-of-life decisions in non-sudden deaths in Belgium, according to community

	Dutch-speaking community		French-speaking community				Belgium total		
	**N**	**%**	**N**	**%**	**OR^b^**	**95%CI**	**N**	**%**	**95%CI^d^**

**Most important end-of life decision that possibly hastened death (ELD)**									
*Number of deaths studied*^a^	*1007*		*637*				*1644*		

Euthanasia/assisted suicide^e^	16	1.6	6	0.9	1.70	[0.66–4.36]	22	1.3	[0.8–2.0]
Administering life-ending drugs without explicit patient request	17	1.7	9	1.4	1.20	[0.53–2.71]	26	1.6	[1.0–2.3]
Intensified alleviation of symptoms	295	29.3	160	25.1	1.21	[0.96–1.53]^c^	455	27.7	[25.5–29.9]
Withholding or withdrawing of life-prolonging treatment	177	17.6	89	14.0	1.33	[0.999–1.77]^c^	266	16.2	[14.4–18.1]

Total ELD	505	50.1	264	41.4	1.43	[1.16–1.77]	769	46.8	[44.3–49.2]
Total ELD with partly or explicitly life-shortening intent	153	15.2	70	11.0	1.42	[1.05–1.93]	223	13.6	[11.9–15.3]

**Continuous deep sedation**^f^									
*Number of deaths studied*^a^	*999*		*630*				*1629*		

All sedation	82	8.2	95	15.1	0.50	[0.37–0.69]	177	10.9	[9.4–12.5]

forgoing artificial food/fluid	36	3.6	37	5.9	0.60	[0.37–0.96]	73	4.5	[3.5–5.6]
administering food/fluid	46	4.6	58	9.2	0.42	[0.28–0.64]	104	6.4	[5.2–7.7]

^a ^Missing data: all questions concerning ELDs unanswered n = 46 (2.7%) (of which 43 cases had died in the hospital: 24 Dutch and 19 French-speaking); question sedation unanswered n = 61 (of which 56 had died in a hospital: 29 Dutch and 27 French-speaking)

^b ^Odds ratios based on logistic regression with community as predictor (French = ref cat) and controlling for patients' age, sex, educational level, cause of death, and place of death

^c ^The probability was significant if alpha = .10 (i.e. level of the CI is 90%)

^d ^Binomial Confidence Interval; exact method

^e ^Physician-assisted suicide was limited to one case

^f ^Sedation was provided in conjunction with any possibly death-hastened decision for 7.9%, and Dutch- and French-speaking communities did not differ.

Controlling for differences in patient age, sex, educational level, cause of death and place of death, the chance of dying following a possibly life-shortening end-of-life decision was higher in the Dutch- (50%) than in the French-speaking community (41%) of Belgium (OR 1.4; 95% CI, 1.2 to 1.8) (Table 2). Selecting all end-of-life decisions with a partly or explicitly life-shortening intention, this difference remained significant (OR 1.4; 95% CI, 1.1 to 1.9). We found higher frequencies for all types of end-of-life decisions with possible life-shortening effect in the Dutch- than in the French-speaking community, but differences did not reach significance at a 95% confidence interval level for any specific type. Euthanasia or physician-assisted suicide (PAS) was performed in 1.6% and 0.9% of cases in the Dutch- and French-speaking community respectively (OR non-significant). Continuous deep sedation both while administering and forgoing food/fluid was registered less often in the Dutch- (8%) than in the French-speaking community (15%) (OR 0.5; 95% CI, 0.4 to 0.7).

Table 3 describes these end-of-life decisions separately for patients who died in the home or care home and for patients who died in a hospital setting. At the home or care home, end-of-life decisions with possible life-shortening effect, especially non-treatment decisions with explicit life-shortening intent, were taken more often in the Dutch- (10%) than in the French-speaking community (5%). Euthanasia/PAS occurred in 1.6% and 1.1% of cases in the Dutch- and French-speaking community respectively (OR non-significant). Continuous deep sedation was performed less frequently in the Dutch- than in the French-speaking homes/care homes (4% versus 8%). Differences for sedation while forgoing food/fluid reached significance at a 90% confidence interval level.

**Table 3:** Frequency of end-of-life decisions in non-sudden deaths according to community and setting in Belgium

**Place of death**	**Home or care home**					**Hospital or palliative care unit**				
	Dutch-speaking		French-speaking			Dutch-speaking		French-speaking		
	**N**	**%**	**N**	**%**	**OR [95%CI]^a^**	**N**	**%**	**N**	**%**	**OR [95%CI]^a^**

Euthanasia/assisted suicide^c^	8	1.6	4	1.1	1.41 [0.42–4.71]	8	1.6	2	0.7	2.28 [0.48–10.82]
Administering life-ending drugs without explicit patient request	10	2.0	7	2.0	1.00 [0.38–2.66]	7	1.4	2	0.7	1.99 [0.41–9.65]
Intensified alleviation of symptoms taking life-shortening into account	155	30.9	95	27.1	1.23 [0.95–1.60]	112	22.1	57	19.9	1.14 [0.80–1.63]
Intensified alleviation of symptoms with partly life-shortening intent	18	3.6	5	1.4	2.47 [1.06–5.75] ^b^	10	2.0	3	1.1	1.90 [0.52–6.97]
Non-treatment taking life-shortening into account	54	10.8	30	8.6	1.30 [0.81–2.11]	31	6.1	12	4.2	1.49 [0.75–2.95]
Non-treatment with explicit life-shortening intent	51	10.2	18	5.1	2.20 [1.24–3.91]	41	8.1	29	10.1	0.78 [0.47–1.29]

Total ELD	296	59.1	159	45.3	1.80 [1.36–2.40]	209	41.3	105	36.7	1.16 [0.85–1.57]

All sedation	22	4.4	29	8.2	0.51 [0.29–0.91]	60	12.0	66	23.7	0.45 [0.30–0.66]
Continuous deep sedation forgoing food/fluid	16	3.2	20	5.7	0.58 [0.33–1.02] ^b^	20	4.0	17	6.1	0.64 [0.33–1.24]
Continuous deep sedation administrating food/fluid	6	1.2	9	2.6	0.45 [0.16–1.29]	40	8.0	49	17.6	0.42 [0.26–0.66]

^a ^Odds ratios based on logistic regression with community as predictor (French = ref cat) and controlling for patients' age, sex, educational level, and cause of death.

^b ^The probability was significant if alpha = .10 (i.e. level of the CI is 90%)

^c ^Cases of euthanasia or assisted suicide were not reported for patients dying in a care home

In the hospital, community differences for end-of-life decisions with possible life-shortening effect were never statistically significant. Continuous deep sedation did occur significantly less frequently in the Dutch- than in the French-speaking community, especially while administering food and fluid (8% versus 18%).

### Discussion with patient

Questions concerning discussion of the decision with the patient remained unanswered in one fifth of cases. In the remaining cases, the decision was discussed in 36% of cases, and 43% of these patients had made an explicit request (Table 4). Seventy-five percent of the patients where no discussion was reported were judged non-competent to make decisions. We found no community differences regarding whether or not the decision was discussed with the patient, whether he/she made an explicit request or whether he/she was non-competent. No data were available concerning the decision-making process of continuous deep sedation. No setting-specific differences between the two communities were found (data not shown).

**Table 4:** Discussion with patient of end-of-life decisions in Belgium, according to community

		Dutch-speaking	French-speaking	Total for Belgium
*Number of deaths studied*^a^		*412*	*191*	*603*

**Discussion with patient**^b^		%	%	%

Administering life-ending drugs without explicit patient request	Discussed	37.5	33.3	36.0
	Not discussed	62.5	66.7	64.0
	Patient non-competent	100.0	83.3	93.8
Intensified alleviation of symptoms	Discussed	30.8	23.3	28.5
	Explicit request	38.6	25.0	35.1
	Not discussed	69.2	76.7	71.5
	Patient non-competent	67.7	67.5	67.7
Withholding or withdrawing of life-prolonging treatment	Discussed	39.9	39.7	39.8
	Explicit request	42.6	41.4	42.2
	Not discussed	60.1	60.3	60.2
	Patient non-competent	88.0	81.8	86.0
Total	Discussed	37.1	32.5	35.7
	Explicit request	45.1	38.7	43.3
	Not discussed	62.9	67.5	64.3
	Patient non-competent	76.3	73.2	75.3

^a ^Missing values for discussion with patient: for all ELDs n = 166 (21%) (93 Dutch and 73 French-speaking) i.e. for administering life-ending drugs without explicit patient request n = 1, for intensified alleviation of symptoms n = 125, for withholding or withdrawing of life-prolonging treatment n = 40.

^b ^**p > .05 **for all characteristics (Fishers' Exact test)

## Discussion

End-of-life decisions with possible, partly or explicitly life-shortening intent occurred in approximately half of all non-sudden deaths in Belgium. Intensified alleviation of symptoms and non-treatment decisions were most prevalent, while euthanasia or physician-assisted suicide (PAS) occurred in 1.3% of cases and life-ending drugs use without explicit patient request in 1.6% of cases. Continuous deep sedation until death occurred in one in ten non-sudden deaths. Although comparing with other studies is difficult due to other research designs, in general, the relatively high occurrence of symptom alleviation, sedation and non-treatment decisions, especially compared to the use of lethal drugs, was also found in previous studies in Belgium, and in several other European countries [3,4,10]. Life-ending drug use without explicit patient request was also found to be relatively high in previous studies in the Dutch-speaking community of Belgium compared to other countries in the world [3,4]. However, we found some remarkable differences between the Dutch- and French-speaking communities in type and prevalence of certain end-of-life decisions, even after controlling for population differences. End-of-life decisions with possible life-shortening effect in general were more prevalent in the Dutch-speaking community, while the practice of continuous deep sedation was more prevalent in the French-speaking community. We could observe a tendency towards higher frequencies of euthanasia/PAS in the Dutch-speaking community, but differences were not significant. Variations between communities did occur according to care setting especially for non-treatment decisions and continuous deep sedation. There were no significant variations in the extent to which decisions were discussed with the patients.

This is the first study on end-of-life decisions in Belgium covering the whole country and the first to describe variations between two language communities in a country where euthanasia is legal. Important strengths of the study include the use of a nationwide and representative surveillance network with a long history of registration research, not selected on the basis of a specific interest in end-of-life research [31,34]; the representativeness of the identified sample of non-sudden deaths; and the quality of the research procedures e.g. weekly registrations could limit memory bias [4,39]. The percentage of non-sudden deaths was also comparable to previous death certificate studies [4].

This study also has limitations. Firstly, GPs were asked to retrospectively indicate whether they or another physician had taken an end-of-life decision. Underestimation is possible especially for patients dying in a hospital and in particular for specific types of decisions which can be seen as part of routine clinical practice and are thus generally less often discussed with other professionals e.g. intensified symptom alleviation [3,18,19]. Results also confirmed that missing data were highest for hospital deaths and for the decision-making process of symptom alleviation. However, it should be noted that a possible underestimation of certain types of decision-making does not make the intra-country comparisons less valid. It does make comparisons with previous incidence studies in Flanders using another research design [3,4] less feasible, especially for these hospital deaths. Secondly, a possible selection or recall bias due to the retrospective design cannot be excluded. Thirdly, due to a low number of cases for some decisions e.g. euthanasia, small differences between groups might not have been identified. Fourthly, the high percentage of missing data concerning the end-of-life decision-making process – probably due to the difficult instructions used – might have biased these results. Fifthly, we did not measure differences in the patient's symptom severity nor in the attitudes of physicians which might have further explained the variations found. We could only control for community differences in patients' age, sex, educational level, cause of death, place of death and possible interaction effects while other possible confounders were not measured. Finally, language was used to differentiate between the two communities of Belgium, but cannot be used to explain the differences we found. Even though language and culture are strongly related, the study of specific underlying cultural factors could have explained the results more fully.

Even though Belgium is a country with one legal and general healthcare system, the higher prevalence of possibly life-shortening end-of-life decisions and lower prevalence of continuous deep sedation in the Dutch-speaking community compared with the French-speaking is remarkable. The difference remained present even after controlling for possible variation in patient characteristics such as age, sex, cause and place of death. Continuous deep sedation is a specialist technique used to relieve intolerable suffering and control refractory distressful symptoms in the last phase of life [14]. Contrary to the other end-of-life decisions where a possible life-shortening effect is always taken into account and sometimes explicitly intended, a life-shortening effect is generally not intended nor expected in cases of continuous deep sedation, except if artificial food and fluid are forgone (i.e. terminal sedation). In these latter cases, death generally is expected and in some cases also intended [14,40,41]. Hence, in the Dutch-speaking community, physicians, patient or their families seem to be more readily prepared to make or ask for life-shortening decisions than in the French-speaking community. In the French-speaking community, they seem to prefer to prolong life for longer, continuing available treatments (including the use of sedative drugs) and to intervene later in the dying process using less direct ways.

This finding corresponds to previous reports on end-of-life care in Belgium. Compared to patients in the Dutch-speaking community, patients in the French-speaking community more often receive possibly life-prolonging treatments and treatment goals are aimed at cure or prolonging life for a longer time in the final three months of life [38]. Additionally, studies from other domains have shown a higher consumption of technological specialist care in the French-speaking part [30]. This stronger appreciation of curative, technological and specialist medicine in the French-speaking community might explain the rarer use of life-shortening decisions and preference towards specialist techniques such as continuous deep sedation. This might be rooted in a general difference in medical culture between the two communities. Since other cross-country studies have shown that life-prolonging treatments (e.g. CPR) and sedative techniques are used more often in southern European countries while possibly life-shortening decisions are more prevalent in several northern European countries [4,14,20,21,26,42,43], the French-speaking community of Belgium seems more closely related to the Latin-French culture in southern European countries, while the Dutch-speaking community corresponds more closely to the northern Germanic European countries.

Therefore, the fact that the public debate on life-shortening and the regulation of euthanasia started early in the Netherlands [44] – lying north of Belgium and where the same language is spoken as in the Dutch-speaking part of Belgium – might have had an early influence on the Dutch-speaking community in Belgium, which was probably absent in the French-speaking community, leading to a higher tendency towards life-shortening decision-making [44].

Concerning euthanasia/PAS, there was a tendency towards a higher prevalence in the Dutch-speaking community but differences were not statistically significant, possibly due to the small number of observed cases. However, actual differences apparently are not large enough to explain the disproportionate percentages observed in the legal notification rate of euthanasia, especially in the home or care home settings [27-29]. Thus, French-speaking physicians, especially general practitioners, seem to report their cases less often to the Federal Evaluation and Control Committee on Euthanasia than the Dutch-speaking. Unfortunately, reasons for this could not be explored. Perhaps French-speaking GPs were less well-informed about the law as the media coverage on euthanasia is higher and training initiatives for physicians are more present in the Dutch-speaking community [45]. Alternatively, culturally determined attitudes towards legal evaluation of medical practices might differ between the two communities. This finding warrants further investigation of French-speaking physicians' attitudes towards the law on euthanasia.

In this study, some interesting setting-specific differences also became apparent. Firstly, analysis showed a higher prevalence of non-treatment decisions with explicit life-shortening effect in the homes/care homes in the Dutch-speaking community but a higher prevalence of continuous deep sedation especially while forgoing artificial food/fluid (terminal sedation) in the French-speaking community, an effect not found in hospitals. Possibly, French-speaking GPs postpone any decision about whether or not a treatment is futile for longer, or let things take their course for a longer time and are then more readily prepared to use sedative drugs instead. Forgoing food/fluid might be a logical consequence of sedative therapy intending to let a patient pass away gently and not a decision primarily intended to shorten life [14,40]. This might partly be explained by the fact that training and consultation initiatives concerning end-of-life decision-making (e.g. communication guidelines, training of physicians to consult colleague physicians) more often originated in the Dutch-speaking community setting in Belgium [45]. Also, preferences for certain end-of-life practices might have a more profound impact in general practice where physicians need to make decisions on their own, compared with hospitals where physicians work within well-defined structures with specific policies regarding complex medico-ethical decisions such as the futility of treatments. However, these hypotheses need more research before definite conclusions can be drawn.

Secondly, continuous deep sedation while administering food/fluid was found to occur noticeably more in hospital settings in the French-speaking community (18% of all non-sudden deaths). Possibly, feeding or hydrating the patient indicates that the physician wants to keep the patient alive [14]. Therefore, the finding might be explained by the strong orientation towards cure in hospital cultures [46] fortified by the general preference for technological curative medicine in the southern community [20,21,30,38,43]. French-speaking hospital physicians seem to reach out for this treatment option sooner or more easily than Dutch-speaking physicians to treat complex problems of terminal patients.

## Conclusion

Even within one country with one legal system and the same basic healthcare options and structures, systematic differences in prevalence and legal reporting of medical end-of-life decisions can occur. Societal and cultural differences between both communities possibly play an important role in determining responses to end-of-life suffering. Further anthropological study is needed to shed light on the mechanisms underlying differences in end-of-life decision-making.

## Competing interests

The authors declare that they have no competing interests.

## Authors' contributions

LVDB conceived the study, acquired, analysed and interpreted the data, drafted the manuscript, participated in obtaining funding for and supervising the study. RD participated in analysing and interpreting the data, critical revision of the manuscript, provided administrative support and co-supervised the study. JB participated in analysing and interpreting the data, critically revised the manuscript for intellectual content and provided administrative, technical, material support. NB participated in data gathering, statistical analysis and interpretation of the data, revision of the manuscript and provision of technical support. VVC was involved in study conception and design, data acquisition, interpretation of the data, manuscript revision, obtaining funding and study supervision. LD conceived the study, was involved in analysis and interpretation of data, manuscript writing, supervision and funding of the study. All authors gave final approval of the manuscript.

## Funding

The first author received a student grant from the Fund for Scientific Research in Flanders, Belgium. Support for the study came from the Research Council of the Vrije Universiteit Brussel in Belgium (project GOA27 2003–2007) and the Institute for the Promotion of Innovation by Science and Technology in Flanders as a Strategic Basic Research project (SBO) (contract SBO IWT 050158) (2006–2010), as part of the 'Monitoring Quality of End-of-Life Care (MELC) Study'. The Belgian Sentinel Network of GPs is supported by the Flemish and Walloon Ministry of Welfare, Public Health and Family. The sponsors had no role in design and conduct of the study, in the collection, analysis, and interpretation of the data, in the writing of the report or in the decision to submit the article for publication.

## Pre-publication history

The pre-publication history for this paper can be accessed here:


